# Estimating the Growth Indices and Nitrogen Status Based on Color Digital Image Analysis During Early Growth Period of Winter Wheat

**DOI:** 10.3389/fpls.2021.619522

**Published:** 2021-04-08

**Authors:** Ben Zhao, Yonghui Zhang, Aiwang Duan, Zhandong Liu, Junfu Xiao, Zugui Liu, Anzhen Qin, Dongfeng Ning, Sen Li, Syed Tahir Ata-Ul-Karim

**Affiliations:** ^1^Key Laboratory of Crop Water Use and Regulation, Ministry of Agriculture, Farmland Irrigation Research Institute, Chinese Academy of Agricultural Sciences, Xinxiang, China; ^2^School of Computer Engineering, Weifang University, Weifang, China; ^3^Institute for Sustainable Agro-Ecosystem Services, The University of Tokyo, Tokyo, Japan; ^4^Department of Global Agricultural Sciences, Graduate School of Agricultural and Life Sciences, The University of Tokyo, Tokyo, Japan

**Keywords:** winter wheat, computer vision technology, nitrogen nutrition index, canopy cover, nitrogen diagnosis

## Abstract

The non-destructive estimation of plant nitrogen (N) status is imperative for timely and in-season crop N management. The objectives of this study were to use canopy cover (CC) to establish the empirical relations between plant growth indices [shoot dry matter (SDM), leaf area index (LAI), shoot N accumulation (SNA), shoot nitrogen concentration (SNC)], and CC as well as to test the feasibility of using CC to assess N nutrition index (NNI) from Feekes 3 to Feekes 6 stages of winter wheat. Four multi-locational (2 sites), multi-cultivars (four cultivars), and multi-N rates (0–300 kg N ha^–1^) field experiments were carried out during 2016 to 2018 seasons. The digital images of the canopy were captured by a digital camera from Feekes 3 to Feekes 6 stages of winter wheat, while SDM, LAI, SNA, and SNC were measured by destructive plant sampling. CC was calculated from digital images developed by self-programmed software. CC showed significant correlations with growth indices (SDM, LAI, and SNA) across the different cultivars and N treatments, except for SNC. However, the stability of these empirical models was affected by cultivar characteristics and N application rates. Plant N status of winter wheat was assessed using CC through two methods (direct and indirect methods). The direct and indirect methods failed to develop a unified linear regression to estimate NNI owing to the high dispersion of winter wheat SNC during its early growth stages. The relationships of CC with SDM, SNC and NNI developed at individual growth stages of winter wheat using both methods were highly significant. The relationships developed at individual growth stages did not need to consider the effect of N dilution process, yet their stability is influenced by cultivar characteristics. This study revealed that CC has larger limitation to be used as a proxy to manage the crop growth and N nutrition during the early growth period of winter wheat despite it is an easily measured index.

## Introduction

Nitrogen (N) is the most important nutritional element for crop growth and its supplemental application is imperative for enhancing crop yield and quality during the crop growth period (Ata-Ul-Karim et al., 2016a, 2017a,b). Chinese farmers generally apply excessive N fertilizer to ensure a higher yield. Previous studies conducted in China reported that the farmer usually applies more than 250 kg N ha^–1^ during the growth season of winter wheat (Zhao et al., 2006). The problem of N over-application not only reduces the income of farmers due to the higher production costs but also increases the risk of environmental pollution (Ata-Ul-Karim et al., 2020a,b). Therefore, the optimization of N fertilizer application of winter wheat is indispensable to reduce over N fertilizer application and to increase environmental sustainability.

Assessing crop N status is the prerequisite for optimizing crop N fertilizer management. Several different indices such as chlorophyll concentration, vegetation index, soil nitrate concentration, shoot N concentration (SNC) have been previously used to indicate crop N status (Lemaire and Gastal, 1997; Zhao et al., 2016, 2018a). SNC is considered as the most direct parameter for reflecting crop N status among the aforementioned indices (Lemaire et al., 2008). The N_*c*_ concentration was proposed and defined as the minimum N concentration required for maximum crop growth by Lemaire et al. (1984). NNI has been considered as a reliable index for diagnosing crop N status and researches conducted in different regions of the world confirmed the reliability of using NNI for crop N status in various crop species (Justes et al., 1994; Yue et al., 2012). The traditional method of NNI calculation requires destructive plant sampling in the field and chemical analysis in the laboratory. The destructive sampling, chemical analysis, and laborious nature of the traditional method of NNI calculation not only make this procedure beyond the expertise of the farmers as it results in failure of provision of timely in-season crop N status owing to the time needed to obtain NNI but also hinders its application as a reliable and accurate tool for scheduling crop N fertilizer management. Consequently, an alternative method is imperative for assessing crop NNI timely and accurately (Lemaire et al., 2008).

Indirect methods such as nitrate concentration in sap, upper layer leaf N concentration, chlorophyll meter readings, and remote sensing tools have been developed to assess NNI to resolve the issues of the traditional NNI calculation method. Justes et al. (1994) reported that when NNI < 1, nitrate concentration in the sap of wheat was never higher than 1 g l^–1^, while for NNI > 1 sap nitrate concentration of wheat can vary from 1 to 10 g l^–1^ irrespective of the NNI value. Nevertheless, this method cannot be used for the quantitative estimation of crop N status. Ziadi et al. (2009) used the N concentration of uppermost collared leaf to assess NNI at the vegetative stage of winter wheat, however, this method also requires destructive sampling for measuring leaf N concentration. Non-destructive chlorophyll estimation and remote sensing technologies are rapid and non-destructive tools to estimate crop N status (Lemaire et al., 2008). Chlorophyll meters have been successfully used to estimate NNI in wheat and maize (Ziadi et al., 2010; Zhao et al., 2018a). However, chlorophyll meter readings were reported to be affected by leaf thickness as readings are generally measured from a small area of leaf 6mm^2^ (Pagola et al., 2009). Besides, leaf measurements with a chlorophyll meter are also time-consuming, which limits their applicability at the field level (Cao et al., 2013; Ata-Ul-Karim et al., 2016b). Remote sensing has been demonstrated as a possibility to quickly assess crop NNI at a larger scale, using sensitive spectral indices (red edge inflection point and normalized difference vegetation index (R_710_, R_512_)) in visible and near-infrared bands (Mistele and Schmidhalter, 2008; Zhao et al., 2018b), but this technology requires the use of high-value equipment (satellite, unmanned aerial vehicle, or field spectrometer) and the professional knowledge to analyze the spectral image and data to produce the reliable results, which limit its commercial application (Wang et al., 2016).

Canopy cover (CC) is a structural parameter that represents the horizontal expansion of the canopy during the vegetative period of the crop. CC is defined as the proportion of cropland covered by the vertical projection of crop canopy (Li et al., 2010). Its measurement methods involved line intersect sampling, modified spherical densitometers, and digital photography (Korhonen et al., 2006). With the development of computer vision technology, the images from the digital camera have been the major method to calculate CC with high accuracy and simplicity for processing the imagery (Li et al., 2010; Nielsen et al., 2012; Lee and Lee, 2013). It only needs to capture an image above the crop canopy and then transmit it to the computer for analysis. Owing to the low-cost and higher efficiency of analyzing CC, the images captured via digital cameras or with smartphones are getting popular among farmers to monitor the status of their crops (Escribano-Rocafort et al., 2014). This method has been used to acquire reliable CC for different crops such as wheat, rice, and maize (Nielsen et al., 2012; Wang et al., 2014; Makanza et al., 2018). The crop canopy image acquired from a digital camera or with a smartphone camera could be used to assess crop actual growth indices (Büchi et al., 2018). CC demonstrated the significant positive correlation with crop growth indices such as leaf area index (LAI), shoot N accumulation (SNA), and SDM during their vegetative growth period (Li et al., 2010; Lee and Lee, 2013), but few studies reported that whether these relationships are stable across different cultivars and N treatments or not. Besides, the increase in CC with the increasing N application rates during the vegetative growth period of rice has also been previously reported (Wang et al., 2016). Combined with the above-mentioned information, this study hypothesized that CC can be considered as a simple and low-cost index to assess crop N status. Therefore, the objectives of this study were to evaluate the stability of the relationships between CC and growth indices (LAI, SNA, SDM, and SNC) across different cultivars and to further explore the possibility of using CC to estimate NNI during early growth period of winter wheat.

## Materials and Methods

### Experimental Design

Four field experiments were carried out during 2016 to 2018 seasons at Xinxiang (35.2°N, 113.8°E) and Qinyang (35.1°N, 112.9°E) using four winter wheat cultivars and varied N rates (0, 75, 150, 225, and 300 kg N ha^–1^). A detailed description of experimental design, soil information, and weather condition are shown in Tables 1, 2. Treatments in all field experiments were arranged using a randomized complete block design with three replicates. The size of each plot was 30m^2^ (6m × 5m) in all experiments. The N fertilizer application was divided into two splits: first before sowing (50%) and second at the Feekes 6 stage (50%). Adequate quantities of triple superphosphate and potassium chloride were applied to the soil before sowing. Winter wheat was over-seeded with hand planters and the thinning was performed to maintain the plant density of 180 × 10^4^ plant ha^–1^ in each experiment. Additional crop management practices were in accordance with the recommendations of the local agriculture department. No obvious water, pest, or disease stress was observed during the wheat growing seasons. The N fertilizer application was the only growth limiting factor.

**TABLE 1:** Basic information about four field experiments conducted during 2016 and 2018 growing seasons at Xinxiang and Qinyang.

**Experiment No.**	**Sowing/Harvesting date**	**Soil characteristics (0–20 cm)**	**Cultivar**	**N (kg N ha^–1^)**	**Sampling stage**
Exp. 1	10-October	Type: light loam soil	Aikang58	0 (N0)	Feekes 3
(2016–2017)	28-May	Organic matter: 10.86 g kg^–1^	(AK58)	75 (N75)	Feekes 5
Xinxiang		Total N: 1.13 g kg^–1^		150 (N150)	Feekes 6
		Olsen-P: 41.47 mg kg^–1^		225 (N225)	
		NH_4_oAc-K^+^: 65 mg kg^–1^		300 (N300)	
Exp. 2	8-October	Type: light loam soil	Bainong207	0 (N0)	Feekes 3
(2017–2018)	30-May	Organic matter: 11.12 g kg^–1^	(BN207)	75 (N75)	Feekes 5
Xinxiang		Total N: 1.24 g kg^–1^		150 (N150)	Feekes 6
		Olsen-P: 48.34 mg kg^–1^		225 (N225)	
		NH_4_oAc-K^+^: 73.94 mg kg^–1^		300 (N300)	
Exp. 3	12-October	Type: light clay soil	Yumai58	0 (N0)	Feekes 3
(2016–2017)	2-June	Organic matter: 13.7 g kg^–1^	(YM58)	75 (N75)	Feekes 5
Qinyang		Total N: 1.22 g kg^–1^		150 (N150)	Feekes 6
		Olsen-P: 64.56 mg kg^–1^		225 (N225)	
		NH_4_oAc-K^+^: 82.36 mg kg^–1^		300 (N300)	
Exp. 4	10-October	Type: light clay soil	Wenmai28	0 (N0)	Feekes 3
(2017–2018)	30-May	Organic matter: 9.8 g kg^–1^	(WM28)	75 (N75)	Feekes 5
Qinyang		Total N: 0.96 g kg^–1^		150 (N150)	Feekes 6
		Olsen-P: 43.87 mg kg^–1^		225 (N225)	
		NH_4_oAc-K^+^: 73.25 mg kg^–1^		300 (N300)	

**TABLE 2:** The weather condition from 2016 to 2018.

**Site**	**Season**	**Average yearly sunshine hours (h)**	**Average yearly precipitation (mm)**	**Average yearly temperature (°C)**
Xinxiang	2016	1945.6	642.3	16.2
	2017	1918.3	663.7	14.3
	2018	1971.9	680.7	15.4
Qinyang	2016	1948.4	578.9	13.7
	2017	1931.7	582.5	14.3
	2018	1966.3	642.5	16.1

### Sampling and Measurement

Plant samples were destructively taken from an area of 0.36 m^2^ at Feekes 3, Feekes 5, and Feekes 6 growth stages of winter wheat (Zadoks et al., 1974) from each plot. The samples were divided into leaves and stems. The green leaf area and LAI were measured using LI-3000 meter (LI-COR). Samples were dried at 105°C for half an hour and then at 70°C until constant weight. The dried samples were weighed to determine SDM and to measure SNC using the Kjeldahl method. The 0.2 g of each sampling was measured using electronic scales. A wet digestion of samples was performed in concentrated sulphuric acid for 4h at 400°C using the Kjeldahl method. The copper sulfate was used as the catalyst in digestion process. The SNA was calculated by multiplying SDM and SNC.

The canopy images of winter wheat were captured on the same day of plant sampling using a digital color camera (D7000, Nikon Inc., Tokyo Japan). The distance between the camera lens and the top of the canopy was close to 1m. The camera was set at an aperture of f/5.6, ISO of 100, white balance of 4900K, auto exposure, and auto-focus with the flash turned off (Wang et al., 2014). The images were taken between 11:00 to 13:00 under clear sky conditions. All the images were stored in the memory card of the camera using the joint photographic expert’s group (JPEG) format, and imported into the computer for further analysis. The resolution of each image was 4272 × 2848 pixels.

### Calculation of CC Using Digital Images

Canopy cover was calculated as the proportion of land covered by the projection of winter wheat canopy due to the view of the photographed image above the winter wheat canopy. A self-design computer program was developed to achieve this by segmenting the images into the canopy and background portions based on the G-R thresholding method. The calculation process was as follows: (1) the monochrome image that had the G-R value was acquired by subtracting the red channel of the image from the green channel and (2) the threshold value of G-R was set for segmenting the monochrome image. When the G-R value of each pixel was higher than the threshold value, this pixel was sorted as the canopy of winter wheat, otherwise, this pixel was sorted as background (Wang et al., 2012). CC was calculated as the proportion of winter wheat canopy pixels to total image pixels.

### Determination of Nitrogen Nutrition Index

The NNI of winter wheat was calculated based on the N_*c*_ curve of winter wheat. The N_*c*_ curve (*N*_*c*_ = 4.15SDM^–0.38^) developed by Yue et al. (2012) was used in this study. The NNI expression formulation was as follow:

(1) $$N\,N\,I=\frac{S\,N\,C}{N_{\text{c}}}$$

Where the SNC represents the actual shoot N concentration (%) at each sampling stage of winter wheat while the N_*c*_ represents the critical shoot N concentration (%) for the same shoot dry matter at each sampling stage of winter wheat. The N nutrition status of winter wheat was considered optimal when NNI = 1. When NNI > 1, N was in excess, and when NNI < 1 growth of winter wheat growth was limited by N deficiency.

### Statistical Analysis

The analysis was performed according to Steel et al. (1997) using the SPSS ver.18 software package (SPSS Inc., Chicago, IL, United States). The homogeneity test of experiment error was conducted before a combined analysis across the four experiments, and then a combined analysis over locations, seasons, N treatments, and cultivars were carried out by the single factor multivariate analysis of variance method. The significance level of all hypotheses testing was preset at *P* < 0.05.

The regression analysis was used to determine the relationships between CC and growth indices (SDM, LAI, SNA, SNC, and NNI) using an allometric curve. For evaluating the stability of the allometric relationships of CC with growth indices (SDM, LAI, SNA, and SNC) across the different cultivars and N treatments, a method named the comparison of linear regressions was used to compare if these individual allometric relationships can merge into one single line across different cultivars and N treatments (Mead and Curnow, 1983). First, these allometric relationships were transformed into linear models using the logarithmic linearization method. Second, the residual variations (error sum of squares, degree of variation, mean square) about a single regression line with all cultivars or N treatments and individual regression lines with each cultivar or N treatment were calculated, respectively. Third, the variation difference values (error sum of squares, degree of variation) of individual lines about a single line was calculated, the corresponding mean square was calculated by dividing the difference values of the error sum of squares into the different values of degree of variation between the single line and the individual lines. Fourth, the *F* value was calculated by the mean square of the difference of variation of individual lines about a single line divided into the mean square of the sum of residual variations about individual lines, the *F* distribution (*a* = 0.05) was used to test if the significant difference exists between these linear regressions. The detailed calculated process was shown as Appendix A.

Two methods were used in this study to assess NNI CC. The direct method, which involves the direct development of a regression model between NNI and CC from Feekes 3 to Feekes 6 stages of winter wheat using an allometric curve (Eq. 2). The second method, an indirect method is based on the definition of NNI (Justes et al., 1994). The SDM and SNC must be assessed before estimating the NNI in the indirect method (Eq. 3 to 5).

(2) $$N\,N\,I={\text{aCC}}^{\text{b}}$$

(3) $$S\,N\,C={\text{a}}_{\text{s}}\,C\,C^{b_{s}}$$

(4) $$S\,D\,M={\text{a}}_{\text{d}}\,C\,C^{{\text{b}}_{\text{d}}}$$

(5a) $$N\,N\,I=\frac{S\,N\,C}{N_{C}}=\frac{S\,N\,C}{4.15\,{\left(S\,D\,M\right)}^{-0.38}}$$

(5b) S⁢N⁢C4.15⁢(S⁢D⁢M)-0.38=a⁢Cs⁢Cbs4.15⋅(ad⁢C⁢Cbd)-0.38

(5c) $$\frac{{\text{a}}_{s}\,C\,C^{b_{s}}}{4.15\,{\left(a_{d}\,C\,C^{b_{d}}\right)}^{-0.38}}=\left(\frac{a_{s}}{4.15\,a_{d}^{-0.38}}\right)\,C\,C^{b_{s}+0.38\,b_{d}}$$

where a and b are the parameters of the regression model between CC and NNI, a_*s*_ and b_*s*_ are the parameters of the regression model between CC and SNC, a_*d*_ and b_*d*_ are the parameters of the regression model between CC and SDM. The N_*c*_ value was calculated from the N_*c*_ curve (*N*_*c*_ = 4.15SDM^–0.38^) developed by Yue et al. (2012). According to the mathematical transformation of the allometric function (5A to 5C), the relationship between NNI and CC in the indirect method was calculated as :

(6) $$N\,N\,I={\text{a}}^{\prime }\,{\text{CC}}^{{\text{b}}^{\prime }}$$

where a′is $\frac{{\text{a}}_{\text{s}}}{4.15\,{\text{a}}_{d}^{-0.38}}$ and b′is *b*_*s*_ + 0.38*b*_*d*_.

## Results

### Variance Analysis of Canopy Cover, Shoot Dry Matter, Shoot Nitrogen Concentration, Shoot Nitrogen Accumulation, Leaf Area Index, and Nitrogen Nutrition Index

According to the results of the homogeneity test across different indices (*P* > 0.05), the single factor multivariate analysis of variance was carried out across different experiments. The effects of location (L), season (S), and cultivar (C) were non-significant on CC, SDM, SNC, SNA, LAI, and NNI. The effect of N on CC was significant (*P* < 0.05) while the effects of N on SDM, SNC, SNA, LAI, and NNI were also significant (*P* < 0.01) (Table 3). All interaction effects to CC were non-significant. The interaction effects of L × N and S × N were significant (*P* < 0.05) for SDM and LAI, while other interaction effects (L × S, L × C, S × C, L × S × C, S × N × C, L × N × C, L × S × N × C) were non-significant for SDM and LAI. The N related indices (SNC, SNA, and NNI) were significantly affected (*P* < 0.01) by N and all interaction effects related with N (L × N, S × N, N × C, S × N × C, L × N × C, L × S × N × C).

**TABLE 3:** The variance analysis of canopy cover, shoot dry matter, leaf area index, shoot nitrogen concentration, shoot nitrogen accumulation, and nitrogen nutrition index for location, season, nitrogen, cultivars, and their possible interactions.

**Indices**	**Location (L)**	**Season (S)**	**Nitrogen (N)**	**Cultivar (C)**	**L × S**	**L × N**	**L × C**	**S × N**	**S × C**	**N × C**	**L × S × C**	**S × N × C**	**L × N × C**	**L × S × N × C**
CC	ns	ns	*	ns	ns	ns	ns	ns	ns	ns	ns	ns	ns	ns
SDM	ns	ns	**	ns	ns	*	ns	*	ns	ns	ns	ns	ns	ns
LAI	ns	ns	**	ns	ns	*	ns	*	ns	ns	ns	ns	ns	ns
SNC	ns	ns	**	ns	ns	**	ns	**	ns	**	ns	**	**	**
SNA	ns	ns	**	ns	ns	**	ns	**	ns	**	ns	**	**	**
NNI	ns	ns	**	ns	ns	**	ns	**	ns	**	ns	**	**	**

ns represents no significant.

* represents significant at 0.05 probability level.

** represents significant at the 0.01 probability level.

### Growth Trends of Canopy Cover During the Vegetative Stage of Winter Wheat

**FIGURE 1:**
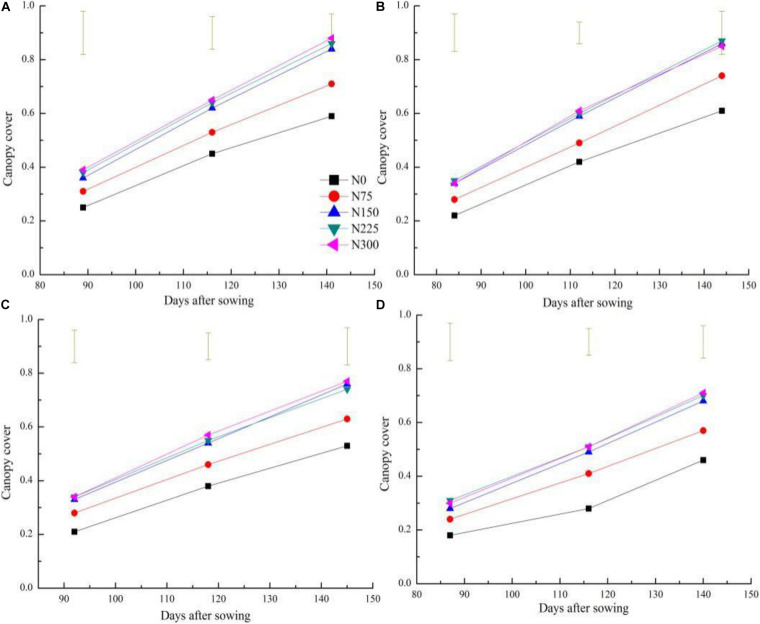
Changes of of canopy cover (CC) across different nitrogen treatments and cultivars during the vegetative stage of winter wheat on 2016–2017 and 2017–2018 seasons **(A)** 2016–2017 AK58; **(B)** 2016–2017 YM58; **(C)** 2017–2018 BN207; **(D)** 2017–2018 WM28. Vertical bars represent the value of least significant difference (*P* < 0.05) for each N treatment.

Canopy cover increased with plant growth and N application rates from Feekes 3 to Feekes 6 stages of winter wheat (Figure 1). The CC of winter wheat ranged from 0.18 ± 0.07 (Feekes 3 stage) to 0.61 ± 0.07 (Feekes 6 stage) and from 0.3 ± 0.05 (Feekes 3 stage) to 0.88 ± 0.04 (Feekes 6 stage) from N0 to N300 treatments, respectively. The growth pattern of the CC under the N limiting treatments demonstrated the same pattern as those as under the non-limiting N treatments at this growth period of winter wheat. However, CC under the non-limiting N treatments was significantly higher than that under the N limiting treatments at the same sampling stage. Yet, minor differences were observed between the CC of the non-limiting N treatments.

### The Relationship Between Canopy Cover and Growth Indices Across Different Cultivars and Nitrogen Treatments

**FIGURE 2:**
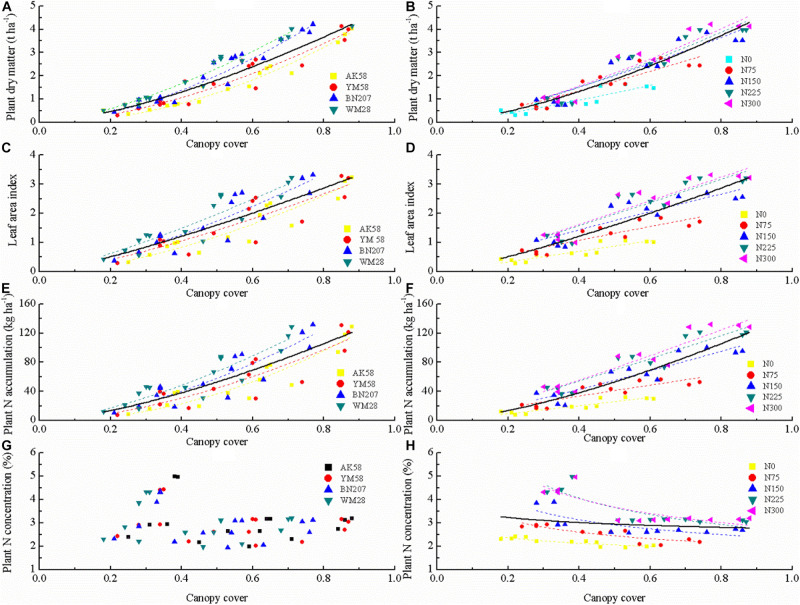
Comparison of the regression relationships of canopy cover (CC) with shoot dry matter (SDM), leaf area index (LAI), shoot nitrogen accumulation (SNA), and shoot nitrogen concentration (SNC) among cultivars and nitrogen(N) fertilization levels. Curves are fitted lines to an allometric function (*Y* = aX^*b*^). **(A)**, **(C)**, **(E)**, and **(G)** denote the relationships between CC and SDM, LAI, SNA and SNC across different cultivars, respectively. **(B)**, **(D)**, **(F)**, and **(H)** denote the relationships between CC and SDM, LAI, SNA and SNC across different N treatments, respectively.

The allometric curve was used to describe CC and growth indices (SDM, SNC, SNA, and LAI) from Feekes 3 to Feekes 6 stages of winter wheat (Figure 2). The significantly positive relationships of CC with SDM, LAI, and SNA were observed among different cultivars and N treatments (Table 4). In contrast, the non-significant relationship between SNC and CC were observed from Feekes 3 to Feekes 6 stages of winter wheat (Figure 2G). Further, according to the comparison result of linear regressions, the allometric relationships of CC with SDM, LAI, and SNA were significantly different among cultivars and N treatments, hence they cannot be pooled together for assessing these growth indices from Feekes 3 to Feekes 6 stages of winter wheat (Table 5).

**TABLE 4:** The model parameters and 95% confidence interval (CI) of the relationships between canopy cover (CC) and growth indices across different cultivars and nitrogen levels.

**Growth Index**	**Cultivar**	**Curve parameter**		**R^2^**	**N level**	**Curve parameter**		**R^2^**
		**a [95% CI]**	**b [95% CI]**			**A [95% CI]**	**B [95% CI]**	
SDM	AK58	4.87 [3.9, 6.08]	1.92 [1.91, 1.93]	0.98**	N0	2.91 [1.51,5.61]	1.24 [1.23,1.25]	0.8**
	YM58	5.05 [3.23, 7.9]	1.76 [1.75,1.77]	0.94**	N75	4.72 [1.42,4.52]	1.44 [1.4,1.42]	0.9**
	BN207	5.79 [4.7, 9.83]	1.79 [1.78, 1.8]	0.96**	N150	5.48 [3.03,9.89]	1.57 [1.55,1.58]	0.91**
	WM28	6.72 [5.06,8.92]	1.54 [1.54,1.55]	0.97**	N225	5.88 [3.43,10.06]	1.64 [1.63,1.65]	0.9**
					N300	5.94 [3.59,9.85]	1.59 [1.58,1.61]	0.91**
LAI	AK58	3.66 [2.07,6.47]	1.63 [1.61,1.64]	0.87**	N0	1.7 [1,2.88]	1 [0.99,1]	0.86**
	YM58	3.68 [1.73,7.82]	1.46 [1.44,1.48]	0.81**	N75	2.68 [1.65,4.34]	1.05 [1.04,1.06]	0.88**
	BN207	4.27 [2.44, 9.3]	1.52 [1.5, 1.54]	0.84**	N150	3.51 [2.15,5.74]	1.07 [1.06,1.08]	0.88**
	WM28	4.95 [2.75,8.93]	1.36 [1.35,1.37]	0.86**	N225	4.27 [2.7,6.75]	1.18 [1.17,1.19]	0.89**
					N300	4.29 [2.75,6.68]	1.14 [1.13,1.15]	0.9**
SNA	AK58	134.37 [64.82,278.57]	1.73 [1.81,1.85]	0.83**	N0	53.62 [30.31,94.89]	1.08 [1.07,1.1]	0.8**
	YM58	134.77 [55.37,328]	1.56 [1.64,1.68]	0.78**	N75	88.5 [49.94,156.84]	1.14 [1.12,1.15]	0.78**
	BN207	176.77 [78.44,398.34]	1.67 [1.64,1.69]	0.78**	N150	127.07 [65.5, 246.52]	1.17 [1.15,1.19]	0.77**
	WM28	185.24 [89.16,384.85]	1.5 [1.48,1.51]	0.81**	N225	154.16 [101.49,231.32]	1.2 [1.18,1.22]	0.88**
					N300	162.06 [110.45,249.77]	1.2 [1.18,1.22]	0.88**
SNC	AK58	–	–	–	N0	1.85 [1.68,2.03]	−0.16 [−0.15, −0.16]	0.68**
	YM58	–	–	–	N75	1.99 [1.69,2.35]	−0.29 [−0.28, −0.29]	0.77**
	BN207	–	–	–	N150	2.38 [1.87,3.03]	−0.29 [−0.28, −0.29]	0.62**
	WM28	–	–	–	N225	2.68 [2.13,3.37]	−0.44 [−0.43, −0.44]	0.76**
					N300	2.78 [2.19, 3.52]	−0.4 [−0.39, −0.41]	0.71**

** represent the significant correlation at the level of *P* < 0.01.

**TABLE 5:** Analysis of variance of comparison of regression models between canopy cover and growth indices across different cultivars and nitrogen treatments.

**Growth index**	**Cultivar**		**N treatment**	
	**F value**	**F_0.05__(__58,52__)_**	**F value**	**F_0.05__(__50,58__)_**
SDM	24.58	1.58	10.43	1.58
LAI	4.48		24.29	
SNA	4.03		30.14	

The curve parameters *a* and *b* of the allometric relationships of CC with SDM, LAI, and SNA were different among four cultivars and five N treatments (Table 4). The values of parameter *a* was higher while the parameter *b* values were lower for cultivar ‘WM28’ as compared to the allometric relationships developed for the other three cultivars. Additionally, the curve parameters *a* and *b* tended to increase with increasing N application rate for the relationships of CC with SDM, LAI, and SNA.

### Changes of Nitrogen Nutrition Index During the Vegetative Stage of Winter Wheat Across Different Nitrogen Treatments

**FIGURE 3:**
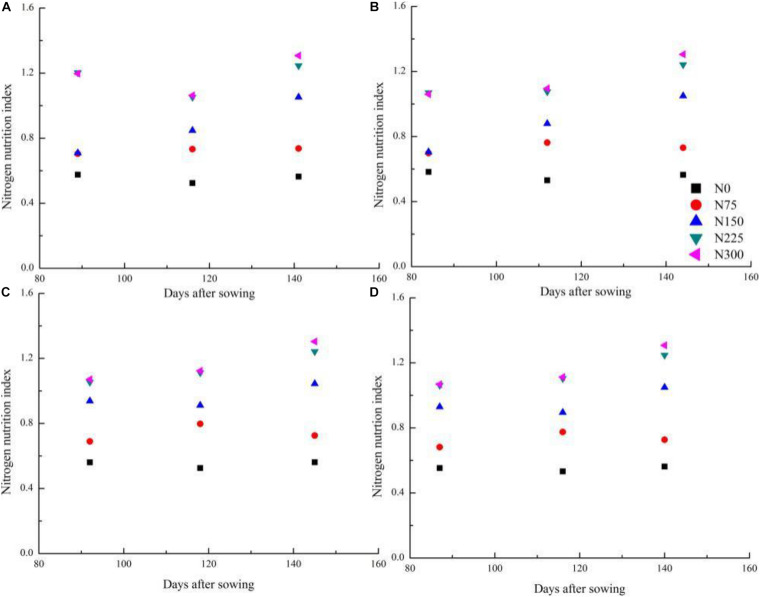
Changes of nitrogen nutrition index during the vegetative period of winter wheat among different cultivars and nitrogen application rates on 2016–2018 growth seasons. [**(A)** 2016–2017 AK58; **(B)** 2016–2017 YM58; **(C)** 2017–2018 BN207; **(D)** 2017–2018 WM28].

Nitrogen nutrition index tended to increase from Feekes 3 to Feekes 6 stages of winter wheat with increasing N supply (N0–N300) during the 2016–2017 and 2017–2018 seasons (Figure 3). The NNI values ranged from 0.52 to 1.31 (Figures 3A,B) and from 0.53 to 1.24 (Figures 3C,D) during the 2016–2017 and 2017–2018 season, respectively. NNI values were less than one for N0 and N75 treatments while under N150 treatment NNI values were close to one. In contrast, NNI values were greater than one for N225 and N300 treatments during the 2016–2017 and 2017–2018 seasons. The change in NNI showed substantial differences across different N treatments and growing seasons.

### Nitrogen Nutrition Index Estimation Model Using a Direct Method

**FIGURE 4:**
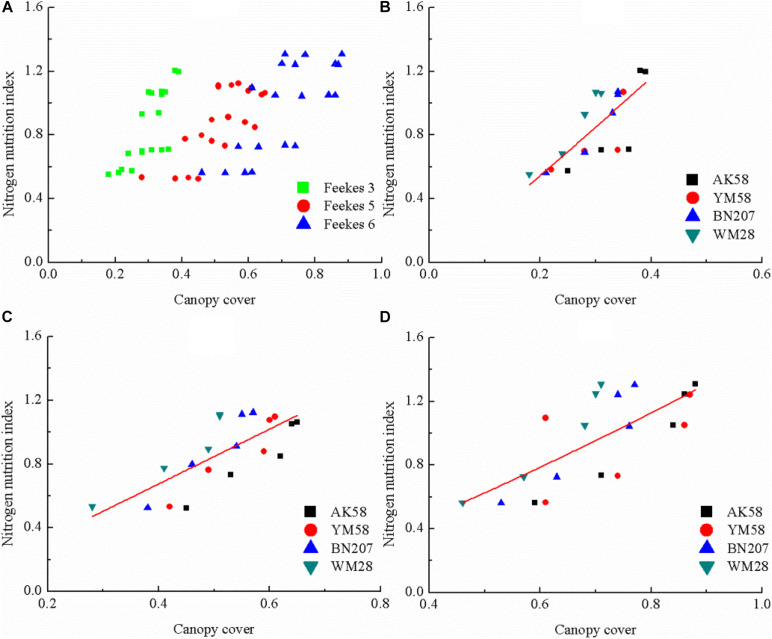
The relationship between canopy cover and nitrogen nutrition index across different growth stages of winter wheat **(A)** the whole vegetative stages; **(B)** Feekes 3; **(C)** Feekes 5; **(D)** Feekes 6.

The allometric relationship between CC and NNI was developed and tested from Feekes 3 to Feekes 6 stages of winter wheat using a direct method. The results indicated a non-significant correlation between NNI and CC when the data were pooled together (Figure 4A), therefore a unified allometric relationship based on CC could not be used for assessing NNI across different vegetative stages of winter wheat. Further, we analyzed the performance of the allometric relationships between NNI and CC across different growth stages (Feekes 3 to Feekes 6 stages), respectively. The results indicated that CC had a significantly positive correlation with NNI at the individual growth stage (Figures 4B–D) having the *R*^2^ values of the allometric relationships greater than 0.55. The direct allometric relationships between CC and NNI at each growth stage was shown in Table 6. The parameter *a* of the relationships between NNI and CC gradually decreased with the growth process of winter wheat, while the parameter *b* showed the opposite trend with that of parameter *a* and increased with the growth process.

**TABLE 6:** The relationships between nitrogen nutrition index and canopy cover across different growth stage using the direct method.

**Growth stage**	**Regression model**	**R^2^**	**Standard error**	**95% CI parameter a**	**95% CI parameter b**
Feekes 3 stage	NNI = 2.92CC^1.04^	0.63**	0.14	[0.76,1.59]	[1,1.07]
Feekes 5 stage	NNI = 1.71CC^1.04^	0.6**	0.14	[0.74,1.61]	[0.99,1.09]
Feekes 6 stage	NNI = 1.53CC^1.38^	0.58**	0.19	[0.8,2.04]	[1.35,1.41]

*The models result from fitting NNI = aCC^*b*^ to our data with the 95% confidence intervals.*

### Nitrogen Nutrition Index Estimation Model Using an Indirect Method

**FIGURE 5:**
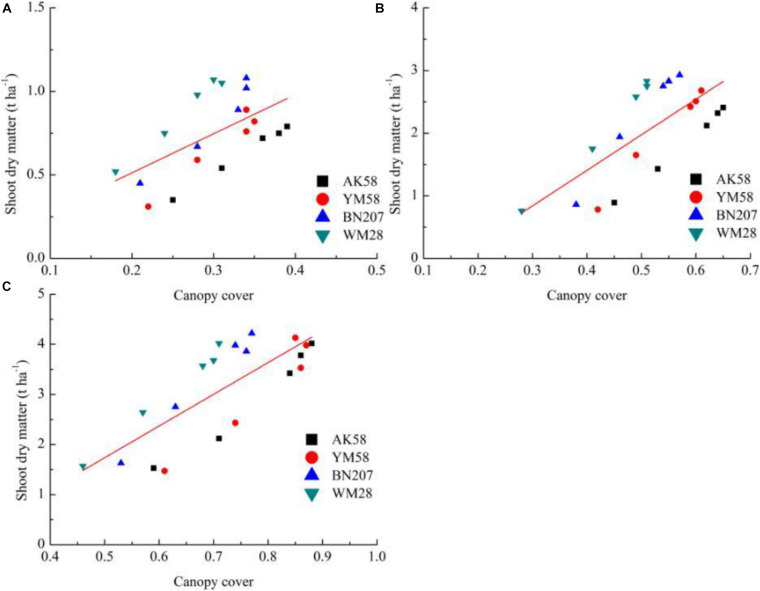
The relationship between canopy cover and shoot dry matter across different growth stages of winter wheat [**(A)** Feekes 3; **(B)** Feekes 5; **(C)** Feekes 6].

**FIGURE 6:**
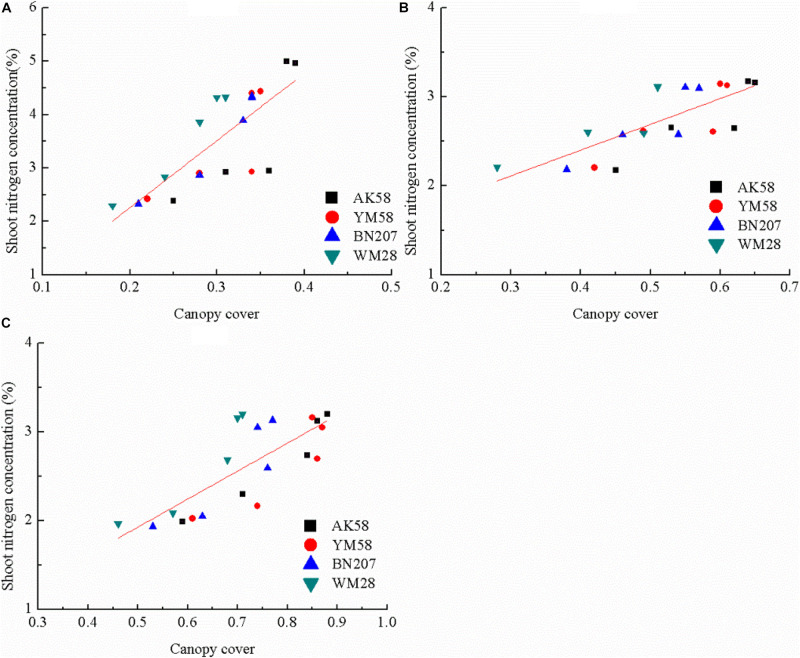
The relationship between canopy cover and shoot nitrogen concentration across different growth stages of winter wheat **(A)** Feekes 3; **(B)** Feekes 5; **(C)** Feekes 6.

Due to the non-significant relationships between CC and SNC across different growth stages (Figure 2G), a unified relationship from Feekes 3 to Feekes 6 stages cannot be developed using the indirect method. Therefore, SNC and SDM at the individual growth stage are required to be estimated using CC for calculating NNI of winter wheat in the indirect method (Figures 5, 6). The results indicated that *R*^2^ values of these relationships were all higher than 0.5. The best relationship was observed at the Feekes 3 stage of winter wheat with *R*^2^ value of 0.65. Similarly, the relationships between SDM and CC were developed using the abovementioned methodology. The results indicated that the relationships between SDM and CC were also positively correlated during the vegetative period of winter wheat. The best relationship was observed at the Feekes 6 stage of winter wheat with *R*^2^ value of 0.63. The estimation models of SNC and SDM were used to calculate NNI indirectly at the individual growth stage of winter wheat. According to Eq. 3 to 6, the indirect estimation model of NNI was shown in Table 7. The final expression pattern of the indirect method to assess NNI was the same (allometric) as that of the direct method (Table 8). In the Feekes 3 and Feekes 5 stages of winter wheat, the model parameters *a* and *b* in the direct method was higher than those in the indirect method while the model parameters *a* and *b* in the direct method was lower than those in the indirect method at the Feekes 6 stage. According to the comparison result of linear regression across different cultivars at the individual growth stage (Table 8), the stability of these linear regressions (SDM and SNC) were still weak, a single regression curve can not well represent the relationship between CC and SDM across different cultivars.

**TABLE 7:** The relationships between nitrogen nutrition index and canopy cover across different growth stages using the indirect method.

**Growth stage**	**Growth indices**	**Regression model**	**R^2^**	**Standard error**	**95% CI parameter a**	**95% CI parameter b**	**Indirect regression**
Feekes 3 stage	SDM	SDM = 2.71CC^1.1^	0.41**	0.19	[1.54,4.75]	[1.04,1.15]	NNI = 2.88CC^1.03^
	SNC	SNC = 11.97CC^1.03^	0.64**	0.58	[8.61,16.43]	[1,1.06]	
Feekes 5 stage	SDM	SDM = 6.2NCC^1.74^	0.59**	0.53	[3.32,11.54]	[1.66,1.82]	NNI = 1.84CC^0.86^
	SNC	SNC = 3.81CC^0.5^	0.56**	0.24	[4.53,9.63]	[0.92,1.02]	
Feekes 6 stage	SDM	SDM = 5.19CC^1.65^	0.63**	0.62	[3.14,8.59]	[1.61,1.69]	NNI = 1.5CC^1.49^
	SNC	SNC = 3.46CC^0.86^	0.64**	0.31	[2.66,4.49]	[0.84,0.88]	

*The models result from fitting SDM = aCC^*b*^ and SNC = aCC^*b*^ to our data with the 95% confidence intervals.*

**TABLE 8:** Analysis of variance of comparison of regression models between canopy cover and growth indices across different cultivars at the different growth stages.

**Growth stage**	**Growth index**	**Cultivar**	
		**F value**	**F_0.05__(__18,12__)_**
Feekes 3	SDM	59.52**	2.6
	SNC	1.72	
Feekes 5	SDM	40.61**	2.6
	SNC	2.38	
Feekes 6	SDM	41.58**	2.6
	SNC	2.89*	

## Discussion

### Changes of Canopy Cover With the Growth of Winter Wheat

Canopy cover of winter wheat increased rapidly when the plant entered into the Feekes 3 stage, which is attributed to the extended leaf length, width, and the increased leaf number with the growth of winter wheat. The expansion rate of CC reached the maximum value at the Feekes 6 stage, where winter wheat plants behaved as isolated and the competition for light between plants remained low and the expansion of CC was not affected with space. The self-shading of leaves and competition for light between plants increased with the expansion of CC and the increasing plant height and resulted in a decline of CC expansion rate after the Feekes 6 stage of winter wheat. There was a different performance of CC across different N treatments. The N application significantly influenced the growth trend of CC in winter wheat. The higher values of CC under non-N limiting treatments than those of N limiting treatments at each sampling stage followed the same response as those of SDM and LAI across different N application conditions (Ata-Ul-Karim et al., 2013; Zhao et al., 2014). Wang et al. (2016) develop a CC-based N_*c*_ curve in rice following the methodology proposed by Justes et al. (1994) for classical plant dry matter-based curves. A similar trend of N dilution based on CC and that based on whole plant SDM indicated that the phenomenon of N dilution is inevitable with the development of crop canopy. It has been widely reported that the N dilution is attributed to the decline in the leaf: stem ratio and the self-shading of leaves during the development of crop canopy (Lemaire et al., 2008).

### The Allometric Relationships Between Canopy Cover and Growth Indices During the Vegetative Period of Winter Wheat

The relationships between CC and growth indices (SDM, LAI, and SNA) from Feekes 3 to Feekes 6 stages of winter wheat in this study were developed using the allometric function. Similarly, Li et al. (2010) and Lee and Lee (2013) used the boolean type function and exponential function to fit the relationships between CC and growth indices in wheat and rice, respectively. In our study, the allometric function was chosen to fit the non-linear relationships between CC and growth indices. The pattern of the allometric function was simple and agronomically sound as compared to other functions. The parameter *a* represents the values of growth indices when CC value was equal to 1, and the parameter *b* is dependent on the accumulation of growth indices in response to CC expansion, which represents the changing trend of growth indices during the crop growth period (Zhao et al., 2017). However, the failure of the allometric curve to assess SNC (Figure 2G) in this study might potentially be attributed to the high dispersion of SNC from Feekes 3 to Feekes 6 stages of winter wheat. According to crop N dilution theory, the inevitable decline of SNC was accredited with crop growth independent of N supplement. Consequently, SNC values of winter wheat were lower at the later growth stages (Feekes 6) than that at the earlier stage (Feekes 3), when CC value was the same across the different N treatments (Figure 2), ultimately resulting in significant variation of SNC with the growth process of winter wheat (Zhao et al., 2017).

The significant positive correlation of CC with SDM, LAI, and SNA (Figures 2A–F) observed in this study was in consensus with previous studies on rice and wheat (Lee and Lee, 2013; Liu et al., 2016). However, the previous studies did not show the effect of cultivars on these relationships between CC and growth indices. According to the comparison result of regression curves (Table 5), the stability of the allometric relationships was weak among different cultivars of winter wheat, the use of a single regression line is significantly worse than the individual fits across different cultivars, the structure and growth characteristic of cultivars might affect the stability of the allometric relationships. The higher parameter *a*_*d*_ of the allometric relationship between CC and SDM at the whole-plant level for WM28 than the other three cultivars indicated the higher rate of SDM accumulation of WM28 during early growth stages. In contrast, the lowest parameter *b*_*d*_ value for WM28 showed that the allocation proportion of SDM to canopy was higher in WM28 as compared to the other three cultivars. The SDM allocated to the canopy resulted in the increased photosynthetic area as a consequence of CC expansion (Mu et al., 2010). This explained the rapid SDM accumulation in WM28. The variation of the parameters *a*_*l*_ and *b*_*l*_ between CC and LAI at the canopy level was similar between CC and SDM across the cultivars. The parameter *a*_*l*_ was the highest and the parameter *b*_*l*_ was the lowest for WM28, which is related to the cultivar structure characteristic. The lower *b*_*l*_ value indicated that the canopy structure characteristic of WM28 (droopier leaf shape with more spreader tillers). Additionally, the allometric relationships between CC and SNA were also developed across different cultivars and confirmed two hypotheses proposed by Lemaire et al. (2007). The first hypothesis was that the N uptake is feed-back governed by crop growth rate. The variations of the parameters *a*_*n*_ and *b*_*n*_ between CC and SNA had very high synchronicity with that of the parameter *a*_*d*_ and *b*_*d*_ between CC and SDM across different cultivars. This synchronicity reflected that SNA capacity seemed to be regulated by crop shoot growth itself. Besides, this regulation by the root transport systems of NO_3_^–^ and/or NH_4_^+^ during the crop growth process has been well documented (Millard, 1988). The second hypothesis was that SNA is often linearly related to the leaf area expansion at the early growth stage of winter wheat. The values of the parameters *b*_*l*_ and *b*_*n*_ were found close for the same cultivar. When the ratio of SNA: LAI was calculated using the model developed based on CC (Table 4), we found that the difference between *b*_*l*_ and *b*_*n*_ values was close to 0. According to the operational rules of the allometric function, the ratio of SNA: LAI was nearly a stable value. The initial slope values ranging between 36.7 and 37.4 kg N ha^–1^ for LAI = 1 across the four cultivars of winter wheat were in consensus with a previously reported slope between SNA and LAI 36.8 kg N ha^–1^ for LAI = 1) for wheat by Lemaire et al. (2007).

There were statistically different relationships of CC with all the growth indices under different N fertilizer treatments. All the growth indices showed an increasing trend with the increase of N fertilizer application. When SDM attained a stable value at the whole plant level, the value of CC under N limiting treatments was higher than that under non-N limiting treatments, which represents that the distribution of SDM is prior to canopy rather than stem under N limiting treatments (Ratjen and Kage, 2016). This allocation pattern increased the photosynthetic area to improve the capacity of photosynthates production. In contrast, self-shading of leaves was more obvious under the non-N limiting treatments as more SDM was allocated to stem to increase plant height for light competition among plants (Lemaire et al., 2019). The CC values under the N limiting treatments were higher than those under the non-N limiting treatments at the same LAI level. This phenomenon further confirmed that the canopy first expanded in the horizontal dimension (length and width) when there is little space competition between plants (Pons et al., 1989). But the competition for light among individual plants under the dense canopy required plants to invest in the vertical dimension (height) to position their leaves to be well illuminated at the top of the canopy (Lemaire et al., 2019). The SNA had a significantly positive relationship with CC at the early growth stage of winter wheat. Owing to the luxury consumption of N, the SNA value was higher under the non-N limiting treatments than under the N limiting treatments even for the same CC value. Crop absorb more N for CC expansion under non-N limiting condition. However, the excessive N might be stored in the lower level of the canopy, which will reduce the difference of N gradient between the upper and lower part of the canopy (Wang et al., 2006).

Based on the above analysis, although the CC applicability for monitoring the growth indices (SDM, LAI, and SNA) was limited by the difference of cultivar characteristic, the parameter difference of the allometric relationship could be used to identify the phenotypic information of cultivars, which contribute to breeding cultivar at the early growth stage of the crop. According to the allometric relationship, the higher parameter *a* and lower parameter *b* represented the higher crop growth and N uptake rate and more compact plant architecture at the same CC condition.

### The Canopy Cover Values as a Predictor of Plant Nitrogen Status

The present study attempted to explore the applicability of the CC parameter to assess and diagnose plant N status at the early growth stages of winter wheat across different N treatments. The CC value was easy to become saturated after the Feekes 6 stage of winter wheat, which limited the application of CC to assess plant N status of winter wheat. Previous reports on oilseed rape and wheat by Behrens and Diepenbrock (2006) and Li et al. (2010) also reported similar results. However, CC can still be used in the N management of winter wheat as N application and scheduling in winter wheat is mainly performed before the Feekes 6 stage (under not saturating conditions, Figure 1).

The measurement of CC using a digital camera followed by computer analysis is an easier, cheaper, and accurate method which is not easily influenced by the environment (Guo et al., 2017). The aforementioned advantages of CC make it seem to be a suitable index to assess crop N status. However, it is disappointing that a unified relationship between the actual CC and NNI was not possible to be developed by pooling the data across different growth stages using both the direct and indirect methods. This was due to two reasons. One reason was that SNC decreases across different growth stages associated with N dilution process (Figure 2G, Lemaire et al., 2019). The other reason was that SNC increases with the increasing N application rate at the individual stage of winter wheat (Figure 1). The two opposite trends caused the high dispersion of SNC during the early growth stages of winter wheat. This dispersion had affected the change of NNI across different growth stages and N treatments and limited the development of the relationship between CC and NNI. On the other hand, CC has a significantly positive relationship with SDM, SNC, and NNI at the individual growth stage of winter wheat, the parameters of these individual linear regressions were different, which further confirmed that unified linear regression is impossible to represent the relationship between CC and NNI across the whole early growth period of winter wheat. Besides, the relatively higher R^2^ values were found between CC and SDM, SNC and NNI at the individual growth stage across the direct and indirect methods (Figures 5, 6). These relationships showed the potential capacity of CC to estimate the growth and N status of winter wheat at the individual stage of winter wheat, but the effect of cultivar characteristics will be considered on the individual linear regression to estimate NNI in further studies.

## Conclusion

This study employed computer vision technology to extract CC and examined the relationships of CC with LAI, SDM, SNA, SNC, and NNI across different cultivars and N treatments during the early growth period of winter wheat. The findings revealed that CC had the potential to assess the growth indices at the individual growth stage of winter wheat, but CC failed to develop a unified linear regression to estimate SDM, LAI, and SNC across different growth stages of winter wheat. Cultivar characteristics affected the development of the relationships between growth indices and CC. The allometric relationships of CC with SDM, LAI, and SNA could show the phenotypic difference in winter wheat. The parameter analysis of these relationships helped to acquire the information of cultivar characteristics during the early growth period of winter wheat. CC had a very obvious limitation in N nutrition management of winter wheat, which was easily affected by the growth and N dilution process. Even if the relationship between CC and NNI was examined at each individual growth stage, the cultivar characteristic would also affect the robustness of the linear regression between CC and SDM, SNC and NNI in the direct and indirect methods. In summary, the CC is an easily obtained and accurate structure index during the growth process of winter wheat, but suffers from certain weaknesses that may limit its use for the estimation of N status in winter wheat.

## Data Availability Statement

The original contributions presented in the study are included in the article/supplementary material, further inquiries can be directed to the corresponding author/s.

## Author Contributions

BZ and SL conceived the idea and led the study design. BZ, SA, and YZ carried out the experiments, performed the analysis, and wrote the manuscript. AD, JX, ZhL, ZuL, and AQ assisted with study design and experiments. DN and YZ edited the manuscript. All authors contributed to the article and approved the submitted version.

## Conflict of Interest

The authors declare that the research was conducted in the absence of any commercial or financial relationships that could be construed as a potential conflict of interest.

## Appendix A

The method of the comparison of linear regressions was used to analyse the difference of linear regression across different cultivars and N treatments. The linear relationship between canopy cover and shoot dry matter across different cultivars was as an example to explain how to compare if the linear regressions merge into a single curve.

Fitted individual regression lines:

**(a) AK58**

*n* = 15

**Table:**

Σ(*y*) = 2.31	Σ(*y*^2^) = 1.86	SSy = 1.51
Σ(*x*) = −4.17	Σ(*x*^2^) = 1.56	SSx = 0.4
	Σ(xy) = 0.13	SSxy = 0.77

*b* = SSxy/SSx = 1.92

*a* = [Σ(y)-bΣ(x)]/*n* = 0.69

Regression s.s. (error sum of squares) = (SS_*xy*_)^2^/S_*xx*_ = 1.48

Residual s.s. = SSyy-regression.s.s = 0.02 (13 d.f.)

**(b) YM58**

*n* = 15

**Table:**

Σ(*y*) = 2.64	Σ(*y*^2^) = 2.1	SSy = 1.63
Σ(*x*) = −4.5	Σ(*x*^2^) = 1.85	SSx = 0.5
	Σ(xy) = 0.08	SSxy = 0.88

*b* = SSxy/SSx = 1.76

*a* = [Σ(y)-bΣ(x)]/*n* = 0.7

Regression s.s. (error sum of squares) = (SS_*xy*_)^2^/S_*xx*_ = 1.54

Residual s.s. = SSyy-regression.s.s = 0.1 (13 d.f.)

**(c) BN207**

*n* = 15

**Table:**

Σ(*y*) = 2.09	Σ(*y*^2^) = 2.19	SSy = 1.37
Σ(*x*) = −5.02	Σ(*x*^2^) = 2.09	SSx = 0.41
	Σ(xy) = −0.45	SSxy = 0.73

*b* = SSxy/SSx = 1.79

a = [Σ(y)-bΣ(x)]/*n* = 0.83

Regression s.s. (error sum of squares) = (SS_*xy*_)^2^/S_*xx*_ = 1.3

Residual s.s. = SSyy-regression.s.s = 0.07 (13 d.f.)

**(d) WM28**

*n* = 15

**Table:**

Σ(*y*) = 3.4	Σ(*y*^2^) = 1.94	SSy = 1.17
Σ(*x*) = −5.84	Σ(*x*^2^) = 2.75	SSx = 0.48
	Σ(xy) = −0.59	SSxy = 0.73

*b* = SSxy/SSx = 1.54

*a* = [Σ(y)-bΣ(x)]/*n* = 0.83

Regression s.s. (error sum of squares) = (SS_*xy*_)^2^/S_*xx*_ = 1.14

Residual s.s. = SSyy-regression.s.s = 0.04(13 d.f.)

**Fitting a single regression line:** If the true relationship is the same for each cultivar then the mean residual variation about a single regression line should be the same as the mean residual variation about separate lines. We therefore fit a single regression line to all 60 points and compare the residual variation about this line with the variation about the four separate lines.

*n* = 60

**Table:**

Σ(*y*) = 8.24	Σ(*y*^2^) = 8.1	SSy = 5.75
Σ(*x*) = −19.52	Σ(*x*^2^) = 8.24	SSx = 1.89
	Σ(xy) = −0.82	SSxy = 3.04

*b* = SSxy/SSx = 1.61

*a* = [Σ(y)-bΣ(x)]/*n* = 0.72

Regression s.s. (error sum of squares) = (SS_*xy*_)^2^/S_*xx*_ = 4.89

Residual s.s. = SSyy-regression.s.s = 0.86(58 d.f.)

**Analysis of variance**

**Table:**

	**s.s**	**d.f.**	**m.s**	**F**
Residual variation about a single line	0.86	58		
Sum of residual variations about individual lines	0.22	52	0.004	
Difference (variation of individual lines	0.64	6	0.11	24.58***
about a single line)				

The calculated *F* value was 24.58. According to the table of F(*a = 0.05*) value, when *v*_1_ = 58 d.f. and *v*_2_ = 52 d.f., the F_0.05_ value is 1.58. The F(24.58) is higher than F(1.58), so the significant difference exist across different linear regression. The use of a single line is significantly worse than the individual fits and we conclude that the relationships are different for four cultivars.
